# Morphometric analysis of the foramen magnum in sex estimation: An additional 3DCT study from Nepal on a larger sample

**DOI:** 10.1002/hsr2.999

**Published:** 2022-12-16

**Authors:** Alok Atreya, Rijen Shrestha, Kiran Bhandari, Saurav K. Malla, Sumnima Acharya, Ritesh G. Menezes

**Affiliations:** ^1^ Department of Forensic Medicine Lumbini Medical College Palpa Nepal; ^2^ Department of Anthropology Punjab University Chandigarh India; ^3^ Department of Radiology Lumbini Medical College Palpa Nepal; ^4^ Department of Radiology & Interventions Grande International Hospital Kathmandu Nepal; ^5^ Department of Pathology, College of Medicine Imam Abdulrahman Bin Faisal University Dammam Saudi Arabia

**Keywords:** forensic anthropology population data, forensic radiology, identification, Nepal, sexual dimorphism

## Abstract

**Background:**

Estimation of sex of the skeletal remains plays a vital part in the identification of an individual. This study is focused on the morphometric measurements of the foramen magnum region and examining the accuracy of sexual dimorphism in the Nepalese population.

**Methods:**

Measurements were obtained from 3D computed tomography (CT) scan of 261 Nepalese adult cranial bases with known age and sex. Length and breadth of the foramen magnum, length and breadth of right and left occipital condyles and maximum and minimum intercondylar distance were measured on the base of the skull CT images.

**Results:**

The mean values for all parameters were higher in males than females except for the maximum intercondylar distance. Sex prediction done with discriminant function analysis could classify the skull with an overall accuracy of 70.5%–71%.

**Conclusions:**

It can be concluded from the results that the morphometric study of the foramen magnum is less reliable for sex estimation in the Nepalese population.

## 1 INTRODUCTION

Identification is of paramount importance in any medicolegal investigation.^1^ Sex estimation is one of the principal indicators in establishing the biological profile of an unknown individual.^2^, ^3^ Sexing of bones becomes easy and accurate when the skeletal remains are intact and complete.^4^, ^5^ However, it is problematic when the bones are fragmented.^6^ Sex estimation is done either by morphoscopic (nonmetric) methods or metric methods.^7^ For the sex estimation from the nonmetric method where the visual assessment is done, the pelvis is considered most dimorphic owing to the morphological changes due to childbirth.^7^ The long bones preferably from the upper limbs are considered more accurate for the metric method of sex estimation where the measurements are obtained from the bone and analyzed using discriminant function and regression equations.^8^, ^9^, ^10^, ^11^ A greater degree of sexual dimorphism is also seen in the skull like mastoid size, gonial angle, size, shape, and so forth, and the skull is considered for both metric and nonmetric methods of sex estimation. Although various methods have been employed for sex estimation in forensic casework there is no absolute cut‐off for reliability as even a random guess is correct 50% of the time.^7^ Therefore, in forensic practice any method which is less than 80% accurate is considered less reliable.^7^

As the base of the skull is made up of thick bony structure and is covered by a large mass of soft tissue, it is well protected during external insults like violence, air accident, arson, explosion, or mass disaster even though the craniofacial structures and other bones are damaged.^12^, ^13^ The foramen magnum is the largest foramen in the human skeleton and is one of the important landmarks of the basicranium.^14^ This has attracted considerable interest among researchers who have undertaken several studies in different populations to determine sexual dimorphism using morphometry.^6^ The use of calipers and calibrated paper strips were used previously to study the morphometry of the cranial base.^13^ Recently, the 3D scans obtained after radiological imaging are gaining popularity. Morphometry is a less expensive method to determine sex with reasonable accuracy, especially in the context of fragmentary remains of victims of mass disasters and skeletonized bodies.^5^

The use of univariate and multivariate functions by anthropologists has suggested that the sexual identity from the cranial samples can be accurately established from 65% to 88%.^13^, ^15^, ^16^, ^17^

There are not many studies pertaining to morphometry and sexual dimorphism in the foramen magnum in the Nepalese population. A previous Nepalese study which was conducted with CT images of the skull bases of 50 male and 50 female subjects calculated the area of the foramen magnum and used discriminant function analysis and predicted sex with 75% accuracy.^18^ This study was conducted to re‐evaluate the morphometry of the foramen magnum along with the occipital condyles for sexual dimorphism by using discriminant function analysis in the Nepalese population by using CT scan images.

## 2 METHODS

The present study was a descriptive cross‐sectional study conducted upon the three‐dimensional computed tomography (3DCT) images of the base of the skull of 261 Nepali individuals whose age and sex were known. All the CT images during the study period were from adults who presented to the Department of Radiology of Lumbini Medical College Teaching Hospital, Palpa for the CT of the head as indicated in the course of their management for diagnostic purposes during the period between February and September 2021.

The study was conducted on normal human skulls of patients (more than 20 years of age for both sexes and with matched ages) who underwent CT scans for various diagnostic medical or surgical indications. Only the high‐quality reconstructed CT images were included and low‐quality blurred images or those with artifacts and those which did not cover the entire area of the foramen magnum were excluded. Noncontrast CT head was done on SIEMENS SOMATOM scope 16 slice CT machine with 0.5 slice thickness and studied with OsiriX MD software. Scans of subjects where there was gross pathology or injury or any deformity in the skull base region were excluded.

**Figure 1:**
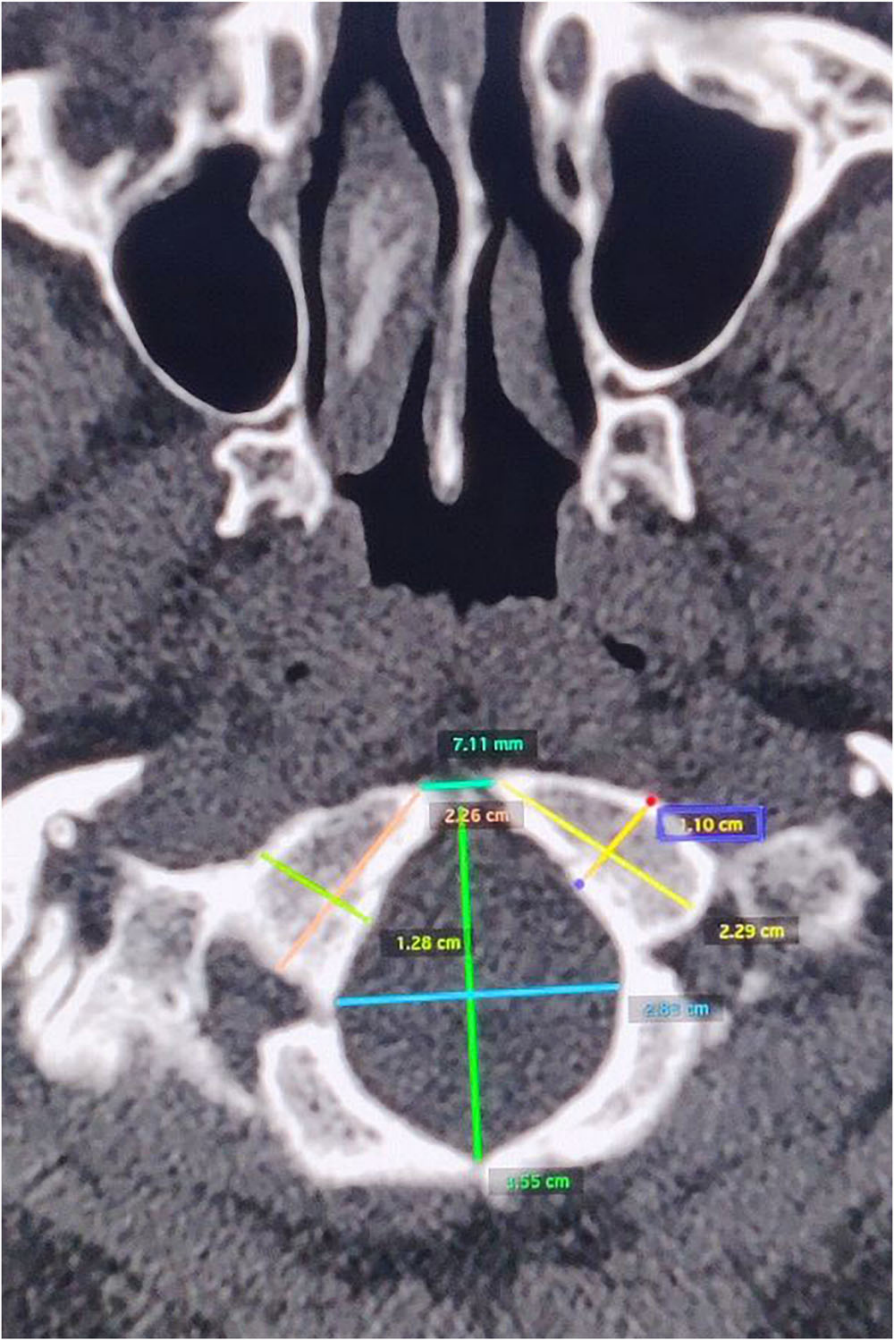
The measurements made in the console of the scanner

For the purpose of the study, 3DCT images were analyzed and various parameters were measured on the console of the scanner on a centimeter scale (Figure 1). Measurements less than 1 cm were expressed in millimeters which were converted to centimeters during data analysis. The parameters that were measured were^14^:

- Foramen magnum length (FML): the distance between the basion and opisthion was taken as the antero‐posterior length of the foramen magnum in the mid‐sagittal plane
- Foramen magnum width (FMW): the distance between the lateral margins of the foramen magnum at the point of the greatest lateral curvature on either side was taken as the transverse diameter of the foramen magnum; perpendicular to the mid‐sagittal plane
- Length of the right occipital condyle (LROC): the distance between the most anterior to the most posterior point on the margin of the right occipital condyle along the long axis
- Length of the left occipital condyle (LLOC): the distance between the most anterior to the most posterior point on the margin of the left occipital condyle along the long axis
- Width of the right occipital condyle (WROC): the distance between the most lateral to the most medial point on the margin of the right occipital condyle perpendicular to the long axis
- Width of the left occipital condyle (WLOC): the distance between the most lateral to the most medial point on the margin of the left occipital condyle perpendicular to the long axis
- Minimum intercondylar distance (MnICD): the distance from the most medial point on the margin of the right and left occipital condyles
- Maximum intercondylar distance (MxICD): the distance from the most lateral point on the margin of the right and left occipital condyles

Two radiologists from the Department of Radiology obtained the data independently. The observers were allowed to manipulate the contrast and brightness features and to use the zoom tool of the software for optimal visualization.

The intra‐ and interobserver reliability was assessed by the intraclass correlation coefficient (ICC). The measurements of the foramen magnum and occipital condyles of nearly 10% of the sample (25 scans) were taken twice by one observer with a time interval of 15 days and once by a second observer. The recalculated measurements were tested, and the ICC values were compared.

The measurements thus obtained were then noted into a proforma and then entered into IBM SPSS Statistics software v 25 and analyzed. The normal distribution of the data was determined by normality plots. The continuous variables were presented as mean ± standard deviation (SD). Male–female differences were tested using the independent *t*‐test and the significance level of the test was defined at *p* < 0.05. Pearson's test was applied to examine the correlation between each foramen magnum dimension in both sexes. The foramen magnum dimensions were further analyzed using discriminate function (DF) analysis using the step‐wise method for sex predictability. The construction of DF was based on the step‐wise method. In a forward selection approach, the best combination of predictors that maximized the (predicted) group classification was identified based on F‐tests.

A cross‐validation approach was based only on those observations that were included in the analysis. An overall measure of accuracy was then presented based on the cross‐validated analysis. Such analysis presents a form of sensitivity analysis and helps to gauge the robustness of the model performance obtained from the final selected model.

Due to the record‐based nature of the study, informed consent was not required in the present study. The study was approved by the Institutional Review Committee of Lumbini Medical College (reference number: IRC‐LMC/12‐J/020). All the underlying data for the present study is available without restriction.^19^

## 3 RESULTS

A total of 261 CT scans from 136 males and 125 females were included in the study. The age ranged from 20 years to 85 years with a mean age of 45.12 ± 17.89 years.

The intra‐ and interobserver reliability was assessed in 25 samples with a two‐way mixed model and absolute agreement, the ICC values of the foramen magnum and occipital condyle measurements were greater than 0.9 except for the maximum intercondylar distance (intraobserver ICC: 0.876, interobserver ICC: 0.853). All the data were found normally distributed on the Q‐Q plot.

The mean values for all measured parameters were higher in males than females except for the maximum intercondylar distance in the present study. The sex differences in the mean values of all the measurements were analysed by the student *t*‐test set at 95% confidence interval and significance set at *p* < 0.05 (Table 1). The results of the *t*‐test showed no significative differences in WROC and MxICD.

**Table 1:** Descriptive statistics of the variables in the foramen magnum region (*n* = 261)

Variable	Sex	Mean	Std. deviation	Std. error	*t* Value	*p* Value
FML	Male	3.48	0.38	0.033	5.519	<0.001
	Female	3.22	0.38	0.034		
FMB	Male	3.01	0.33	0.028	5.890	<0.001
	Female	2.78	0.30	0.026		
LROC	Male	2.27	0.24	0.021	3.210	0.001
	Female	2.18	0.19	0.017		
WROC	Male	1.23	0.16	0.014	0.983	0.326
	Female	1.21	0.14	0.013		
LLOC	Male	2.30	0.27	0.023	3.521	0.001
	Female	2.19	0.22	0.020		
WLOC	Male	1.28	0.25	0.022	2.356	0.019
	Female	1.21	0.15	0.013		
MnICD	Male	1.40	0.24	0.020	3.876	<0.001
	Female	1.29	0.21	0.019		
MxICD	Male	4.50	0.74	0.063	−0.028	0.978
	Female	4.51	0.57	0.051		

Abbreviations: FML, foramen length; LLOC, length of the left occipital condyle; LROC, length of the right occipital condyle; MnICD, minimum intercondylar distance; MxICD, maximum intercondylar distance; WLOC, width of the left occipital condyle; WROC, width of the right occipital condyle.

The correlation between all the variables was examined using Pearson's test for both sexes as shown in Tables 2 and 3. In both the sexes a strong correlation was seen in FML/FMW (*r* = 0.676 in males; *r* = 0.618 in females). In males, the strongest correlation was observed in LROC/LLOC (*r* = 0.759) and in females, strongest correlation was observed in WROC/WLOC (*r* = 0.743). The strongest negative correlation was observed in FML/MxICD in both males and females.

**Table 2:** Correlation between all variables independently in males (*n* = 136)

Variables		FML	FMW	LROC	WROC	LLOC	WLOC	MnICD	MxICD
FML	r	1	0.676**	0.186*	0.242**	0.085	0.128	0.132	−0.194*
	p		<0.001	0.030	0.005	0.324	0.139	0.126	0.024
FMW	r		1	0.094	0.064	−0.086	0.108	0.332**	−0.142
	p			0.278	0.457	0.322	0.212	<0.001	0.098
LROC	r			1	0.431**	0.759**	0.179*	−0.034	0.363**
	p				<0.001	<0.001	0.037	0.690	<0.001
WROC	r				1	0.357**	0.475**	−0.012	0.344**
	p					<0.001	<0.001	0.887	<0.001
LLOC	r					1	0.130	−0.173*	0.294**
	p						0.132	0.044	<0.001
WLOC	r						1	−0.010	0.303**
	p							0.907	<0.001
MnICD	r							1	0.024
	p								0.781
MxICD	r								1
	p								

Abbreviations: FML, foramen length; LLOC, length of the left occipital condyle; LROC, length of the right occipital condyle; MnICD, minimum intercondylar distance; MxICD, maximum intercondylar distance; WLOC, width of the left occipital condyle; WROC, width of the right occipital condyle.

** Correlation is significant at the 0.01 level (2‐tailed).

* Correlation is significant at the 0.05 level (2‐tailed).

**Table 3:** Correlation between all variables independently in females (*n* = 125)

Variables		FML	FMW	LROC	WROC	LLOC	WLOC	MnICD	MxICD
FML	r	1	0.618**	0.155	0.166	0.001	0.134	0.123	−0.248**
	p		<0.001	0.084	0.064	0.989	0.137	0.173	0.005
FMW	r		1	0.096	−0.073	0.011	0.009	0.144	−0.157
	p			0.286	0.416	0.905	0.918	0.110	0.080
LROC	r			1	0.411**	0.608**	0.324**	0.001	0.462**
	p				<0.001	<0.001	<0.001	0.993	<0.001
WROC	r				1	0.275**	0.743**	0.043	0.348**
	p					0.002	<0.001	0.632	<0.001
LLOC	r					1	0.374**	−0.027	0.411**
	p						<0.001	0.762	<0.001
WLOC	r						1	0.133	0.401**
	p							0.139	<0.001
MnICD	r							1	−0.129
	p								0.152
MxICD	r								1
	p								

Abbreviations: FML, foramen length; LLOC, length of the left occipital condyle; LROC, length of the right occipital condyle; MnICD, minimum intercondylar distance; MxICD, maximum intercondylar distance; WLOC, width of the left occipital condyle; WROC, width of the right occipital condyle.

** Correlation is significant at the 0.01 level (2‐tailed).

The variables were then analysed for discriminant function using both univariate and multivariate methods (Table 4). The hypothesis testing of the multivariate data was done with Wilks's Lambda with a F‐to‐enter value of 3.84 and a F‐to‐remove value of 2.71. Wilk's Lambda was determined to be 0.820 which rejected the null hypothesis—the mean score of all the variables in both males and females were equal (i.e., sexual dimorphism does not exist). The canonical discriminant function was 0.424, eigen value 0.248, and the group centroid was 0.447 for males and −0.487 for females with a cut‐off score of −0.02. When a step‐wise method was adopted, the three variables FMW, LLOC, and MnICD showed higher classification accuracy (Table 5).

**Table 4:** Canonical discriminate coefficients, percentages of correct group membership and cross validation of craniometric measurements (*n* = 261)

Variables	Eigen value	Wilk's lambda	Canonical correlation	Fisher's linear DF		Group centroid		Prediction after cross validation^a^ (%)		
				Male	Female	Male	Female	Male	Female	Overall
Univariate analysis										
FML	0.117	0.895	−8.731	−41.715	−35.759	0.327	−0.356	63.2	63.2	63.2
FMW	0.133	0.883	−9.062	−44.964	−38.394	0.348	−0.378	70.6	64.0	67.3
LROC	0.39	0.962	−9.993	−52.525	−48.592	0.189	−0.205	52.2	65.6	59.0
WROC	0.004	0.996	−7.843	−31.910	−30.958	0.058	−0.063	46.3	58.4	52.3
LLOC	0.47	0.955	−8.902	−42.184	−38.338	0.207	−0.225	54.4	60.8	57.6
WLOC	0.21	0.980	−5.821	−18.440	−16.776	0.137	−0.149	47.1	60.8	53.9
MnICD	0.057	0.946	−5.837	−19.093	−16.309	0.229	−0.249	57.4	68.8	63.1
MxICD	0.000	1	−6.771	−23.603	−23.626	−0.002	0.002	39.7	64	51.8
Multivariate analysis										
FML	0.248	0.802	0.098	1.171	1.074	0.475	−0.517	68.4	73.6	71
FMW			0.124	1.929	1.807					
LROC			−0.001	0.664	0.665					
WROC			−0.165	1.510	1.673					
LLOC			0.206	2.414	2.209					
WLOC			0.112	0.066	−0.044					
MnICD			0.167	2.078	1.912					
MxICD			−0.002	0.798	0.800					
Constant			−13.033	−127.893	−114.982					

Abbreviations: FML, foramen length; LLOC, length of the left occipital condyle; LROC, length of the right occipital condyle; MnICD, minimum intercondylar distance; MxICD, maximum intercondylar distance; WLOC, width of the left occipital condyle; WROC, width of the right occipital condyle.

^a^ Cross validation is done only for those cases in the analysis. In cross validation, each case is classified by the functions derived from all cases other than that case.

**Table 5:** Discriminant function analysis using step wise method

Step	Variables entered	Wilks' lambda^a^				Fisher exact			
		Statistics	Df1	Df2	Df3	Statistics	Df1	Df2	Sig.
1	FMB	0.883	1	1	259	34.356	1	259	<0.001
2	FMB	0.842	2	1	259	24.193	2	258	<0.001
	LLOC								
3	FMB	0.820	3	1	259	18.797	3	257	<0.001
	LLOC								
	MnICD								

Abbreviations: LLOC, length of the left occipital condyle; LMnICD, minimum intercondylar distance; MxICD, maximum intercondylar distance.

^a^ At each step, the variable that minimizes the overall Wilks' Lambda is entered.

Cross‐validation using a jack‐knifed approach that is, for each observation, sex classification is obtained by using a function constructed from the remaining observations. This means that for each of the observations in the data set (*N* = 261), the classification (discriminant) function was derived using data from the remaining 260 observations. Based on the discrimination function analysis, the classification of the sex was estimated. Canonical discriminant function coefficients of the selected variables and sex prediction accuracy based upon these variables are presented in Table 6. The results show that the function provides an overall accuracy of 70.5% during sex estimation.

**Table 6:** Discriminant function coefficient of selected variables and the classification statistics

Canonical DF (unstandardized)	Function	Predicted group membership by cross validation^a^ (%)		
	1	Male	Female	Overall
Foramen magnum breadth	0.220	69.9	71.2	70.5
Lt occipital condyle length	0.214			
Minimum intercondylar distance	0.171			
(Constant)	−13.505			

Abbreviation: DF, discriminate function.

^a^ Cross validation is done only for those cases in the analysis. In cross validation, each case is classified by the functions derived from all cases other than that case.

## 4 DISCUSSION

The foramen magnum is chosen to identify the sex of partial or damaged skeleton in forensic caseworks. This is because it is a regular structure that is unlikely to undergo significant morphological changes. Its development can be used to describe the degree of sexual dimorphism exists within the dimensions of foramen magnum. Compared to many other skeletal components, the foramen magnum matures relatively early in childhood and is unlikely to respond significantly to secondary sexual changes. The foramen magnum is not controlled by muscles; rather, it serves as a passageway for structures entering and exiting the cranial base region, particularly the medulla oblongata, which occupies the majority of the foraminal space. Because the nervous system is the most precocious of all body systems, it matures at a very young age and does not need to grow in size. The occipital bone fuses completely by the time a child is 5 to 7‐year‐old.^13^

In 1967, Radinsky measured the foramen magnum length and breadth to calculate the area of the foramen magnum which he utilized to approximate the brain size in mammals.^20^ In 1982, Teixeira measured the length and breadth of the foramen magnum of 40 exhumed skulls and stated that the area was larger in males than in females.^21^ Teixeira cited the books by Fatteh (1973), Krogman (1978), and Keen (1978) and stated that his findings were similar to that of the authors.^21^ Radinsky's and Teixeira's methods are based upon the calculation of the area of the foramen magnum considering it as a circle and it is widely used by researchers across the globe for sex estimation. The shape of the foramen magnum is not always round and there are various shapes like oval, tetragonal, pentagonal, hexagonal, egg, and irregular.^22^ Differences are observed when calculating the area of the foramen magnum by Radinsky's method, Teixeira's method, and while using computer software.^22^, ^23^

Similar to the previous studies, the present study also found that the mean values of various metric measurements were greater in males than in females. Sexual dimorphism could be established from the foramen magnum which was 71% by multivariate analysis and 70.5% for selected variables who showed higher classification accuracy in step wise method in the present study. The classification accuracy ranged from 51.8% to 67.3% based on the univariate analysis. When the results of univariate and multivariate analysis were compared, it was clear that multivariate analysis outperformed the former in terms of both reliability and accuracy. Therefore, multivariate analysis is by far the best technique for estimating sex of the skull. Some of the studies conducted across the world show a sex prediction accuracy of 50.2%–90% (Table 7).^1^, ^2^, ^4^, ^6^, ^12^, ^13^, ^14^, ^15^, ^16^, ^18^, ^24^, ^25^, ^26^, ^27^, ^28^, ^29^, ^31^, ^32^, ^33^, ^34^, ^35^, ^36^, ^37^, ^38^, ^39^, ^40^

**Table 7:** Sex prediction accuracy from the foramen magnum region across various studies

Geographical region	Sample population (age range)	Authors	Sample size	Anatomical region	Method	Sex prediction
South Asia	Indian	Raghavendra Babu et al.^24^	90 (♂−50, ♀−40)	FM	Postmortem–dry skull	88%
	Indian (25–65 years)	Raikar et al.^25^	150 (♂−75, ♀−75)	FM	Antemortem–digital radiograph image	67.3%
	Indian (>18 years)	Kamath et al.^26^	72 (♂−41, ♀−31)	FM	Postmortem–dry skull	70.3%
	Indian (20–80 years)	Jaitley et al.^27^	280 (♂−140, ♀−140)	FM	Antemortem–CBCT image	72%
	Indian	Vinutha et al.^28^	200 (♂−110, ♀−90)	FM	Antemortem–CT image	65%
	Indian (18–70 years)	Tambawala et al.^29^	266 (♂−111, ♀−115)	FM	Antemortem–CBCT image	66.4%
	Nepalese (15–85 years)	Singh et al.^18^	100 (♂−50, ♀−50)	FM	Antemortem–CT image	75%
	Nepalese (20–65 years)	Maharjan et al.^30^	96 (♂− 53, ♀−43)	FM	Postmortem–skull at autopsy	75%
	Nepalese (20–85 years)	Present study	261 (♂− 136, ♀− 125)	FM and OC	Antemortem–CT image	70.55%
North Africa and Middle East	Iraqi (20–49 years)	Uthman et al.^31^	88 (♂−43, ♀−45)	FM	Antemortem–helical CT image	81.8%
	Turkish (21–50 years)	Meral et al.^32^	600 (♂−300, ♀−300)	FM	Antemortem–CT image	75%
	Saudi Arabian (20–70 years)	Madadin et al.^1^	200 (♂−100, ♀−100)	FM and OC	Antemortem–CT image	71%
	Saudi Arabian (18–72 years)	Aljarrah et al.^33^	472 (♂−236, ♀−236)	FM and OC	Antemortem–CT image	71.6%
	Iranian (15–50 years)	Hosseini et al.^34^	229 (♂−120, ♀−109)	FM	Antemortem–CT image	50.2%
	Turkish (19–88 years)	Tellioglu et al.^35^	100 (♂−50, ♀−50)	FM	Antemortem–CT image	67%
Europe	European (18–85 years)	Ilguy et al.^36^	161 (♂−66, ♀−95)	FM and mandible	Antemortem–CBCT image	83.2%
	Modern Greek (19–99 years)	Chovalopoulou et al.^16^	154 (♂−77, ♀−77)	FM and OC	Postmortem–dry skull	74%
	Southern Spanish (22–93 years)	Amores‐Ampuero^15^	109 (♂−53, ♀−56)	FM and OC	Postmortem–dry skull	75.7%
	18th–19th century British (18–90 years)	Gapert et al.^13^	158 (♂−82, ♀−76)	FM	Postmortem–dry skull	68%
	18th–19th century skeletal remains, St. Bride's church, London (18–90 years)	Gapert et al.^12^	135 (♂−69, ♀−66)	FM and OC	Postmortem–dry skull	71.9%
	Portuguese juvenile born between 1805 and 1972 (8–19 years)	Veroni et al.0^37^	36 (♂−17, ♀−19)	FM and OC	Postmortem–dry skull	75.8%
	Bulgarian adults (♂−61.5 ± 15.5 years, ♀−64.4 ± 14.3 years)	Toneva et al.^6^	140 (♂−70, ♀−70)	FM	CT image	74.9%
Americas	American (20–50 years)	Holland^38^	120 (♂−60, ♀−60)	FM	Postmortem–dry skull	71%–90%
	American (20–80 years)	Wescott et al.^39^	389 white, 133 black	FM and OC	Postmortem–dry skull	76%
	Brazilian (18–104 years)	Lopez‐Capp et al.^2^	100 (♂−53, ♀−47)	FM	Postmortem–dry skull	60%–65%
	Colombian (18–50 years)	Gonzalez‐Colmenares et al.^4^	115 (♂−71, ♀− 44)	FM	Postmortem–skull base radiograph	85.7%–87.2%
	Brazilian (♂−44.3 ± 12.9 years, ♀−40.76 ± 16.7 years)	Suazo et al.^40^	211 (♂−144, ♀−71)	FM	Postmortem–dry skull	66.5%

The highest sex prediction was made by Holland who used a regression equation upon nine measurements from 100 crania and claimed the accuracy up to 90%.^38^ Wescott et al.^39^ in 2001, measured the foramen magnum and occipital condyles of 389 skulls which also included the samples used by Holland in 1986, and found several discrepancies in the measurements obtained by Holland. Westcott et al.^39^ could predict sex with only 76% accuracy in the samples measured by Holland who reported up to 90% accuracy.

Seifert et al.^3^ discuss various reasons for limited applicability of using foramen magnum region for sex estimation‐ the first being the reference sample “which are often outdated as referential to contemporary populations due to secular trends, which themselves greatly influence metric features observed in populations during time” (p. 126.e7) and secondly the accuracy and reliability of the method used. Since sex is a discrete dichotomous variable (male or female), there is a 50% chance that it will be properly predicted at random.^2^ It is not practical to rely upon one trait or one isolated structure to estimate accurately the sex of the entire skeleton although statistically significant differences are observed.^3^, ^41^ Although the findings of the present study show that the region of the foramen magnum is sexually dimorphic and statistically significant differences were observed, it should be cautiously used for sex estimation in forensic work because the sex classification accuracy in the Nepali population is 70.55%–71%.

## AUTHOR CONTRIBUTIONS

**Alok Atreya**: Formal analysis; investigation; methodology; resources; visualization; writing – original draft; writing – review & editing. **Rijen Shrestha**: Conceptualization; formal analysis; visualization; writing – review & editing. **Kiran Bhandari**: Data curation; methodology; writing – original draft. **Saurav Krishna Malla**: Formal analysis; methodology; writing – original draft. **Sumnima Acharya**: Data curation; Methodology; writing – review & editing. **Ritesh G Menezes**: Conceptualization; supervision; writing – review & editing.

## CONFLICT OF INTEREST

The authors declare no conflict of interest.

## ETHICS STATEMENT

Due to the record‐based nature of the study, informed consent was not required in the present study. The study was approved by the Institutional Review Committee of Lumbini Medical College (reference number: IRC‐LMC/12‐J/020).

## TRANSPARENCY STATEMENT

The lead author Alok Atreya affirms that this manuscript is an honest, accurate, and transparent account of the study being reported; that no important aspects of the study have been omitted; and that any discrepancies from the study as planned (and, if relevant, registered) have been explained.

## Data Availability

The data that support the findings of this study are openly available in “Dryad” at doi:10.5061/dryad.5qfttdz8w.^19^
